# Massive hemoptysis in a respiratory ICU: causes, interventions and outcomes - Indian study

**DOI:** 10.1186/cc10688

**Published:** 2012-03-20

**Authors:** D Talwar, J Chudiwal, R Jain, S Kumar

**Affiliations:** 1 Metro Center for Respiratory Diseases, Noida, India

## Introduction

Massive hemoptysis carries high mortality and morbidity, requiring multidisciplinary management. In India, tuberculosis is a very common cause of severe hemoptysis and is being treated in tuberculosis hospitals where such an approach is not available. We evaluated the profile of patients admitted with massive hemoptysis in a well-equipped Indian tertiary-care respiratory center.

## Methods

Retrospective analysis of 376 patients admitted with hemoptysis to the respiratory ICU of the Metro Center for Respiratory Diseases, India was done. We identified 90 patients with massive hemoptysis (>600 ml in 24 hours) between 2005 and 2011 and the results were analyzed. As per our protocol all patients had active medical management and those suitable for surgery underwent elective or emergent surgery. Unsuitable candidates underwent bronchial artery embolisation (BAE) or bronchoscopic interventions (BI) and if suitable were taken for surgery later.

## Results

The mean age of patients was 49.5 ± 16.53 years with 73.33% (*n *= 66) being male. Mortality in male patients was significantly higher than females (64.7 vs. 35.3%, *P *= 0.02). The mean length of stay in hospital was 10.44 ± 6.9 days and significantly less (7.06 ± 4.8, *P *= 0.01) in the mortality group. Massive hemoptysis was due to tuberculosis (active and old) in 61%, pneumonia in 25.5%, bronchiectasis in 21.1%, aspergillus-releated disease in 11.1%. Lung cancer in 6.6% cases but this carried highest mortality. The bleeding site was identified on CT chest in 65.5% and in 64.4% by fiber optic bronchoscopy (FOB). However, combined FOB and CT scan could localize bleeding in 87.8%. See Table 1.

**Table 1:** Types of management versus mortality in massive hemoptysis

Management	Total	Mortality	Percentage
Medical	6	5	83.3
BI	35	7	20
BAE	19	1	5.2
Surgery	30	4	13.3
Multiple	21	2	9.5

## Conclusion

All-cause mortality in massive hemoptysis at our center was 18.8%. Lung cancer, necrotizing pneumonia and bronchiectasis carried significantly higher mortality. BAE showed low mortality but required multiple interventions in nearly two-thirds of cases. Hence, surgery remains the intervention of choice in massive hemoptysis at our setup with acceptable mortality and outcome.

